# Characterization of Depressive Symptoms Trajectories After Breast Cancer Diagnosis in Women in France

**DOI:** 10.1001/jamanetworkopen.2022.5118

**Published:** 2022-04-14

**Authors:** Cécile Charles, Aurélie Bardet, Alicia Larive, Philip Gorwood, Nicolas Ramoz, Emilie Thomas, Alain Viari, Marina Rousseau-Tsangaris, Agnès Dumas, Gwenn Menvielle, Sibille Everhard, Anne-Laure Martin, Seyive-yvon-arnauld Gbenou, Julie Havas, Mayssam El-Mouhebb, Antonio Di Meglio, Fabrice André, Barbara Pistilli, Charles Coutant, Paul Cottu, Asma Mérimèche, Florence Lerebours, Olivier Tredan, Laurence Vanlemmens, Christelle Jouannaud, Christelle Levy, Ines Vaz-Luis, Stefan Michiels, Sarah Dauchy

**Affiliations:** 1 Department of Prevention-Public Health, Institut Bergonié, Bordeaux, France; 2 Bordeaux Population Health, Institut National de la Santé et de la Recherche Médicale (INSERM) U1219, Université de Bordeaux, Bordeaux, France; 3 Gustave Roussy, Université Paris-Saclay, Biostatistics and Epidemiology Office, Villejuif, France; 4 Oncostat U1018 INSERM, University Paris-Saclay, Ligue Contre le Cancer, Villejuif, France; 5 Institute of Psychiatry and Neuroscience of Paris, INSERM U1266, Université de Paris, Paris, France; 6 La Clinique des Maladies Mentales et de l'Encéphale, Le Groupe Hospitalier Universitaire Paris Psychiatrie et Neurosciences, Hôpital Sainte-Anne, Paris, France; 7 Fondation Synergie Lyon Cancer Plateforme Bioinformatique Gilles Thomas, Lyon, France; 8 Épidémiologie Clinique et Évaluation Économique Appliquées aux Populations Vulnérables, Unité Mixte de Recherche 1123 INSERM, Université de Paris, Paris, France; 9 Unicancer, Paris, France; 10 Gustave Roussy, INSERM U981, Université Paris-Saclay, Villejuif, France; 11 Centre Georges-François Leclerc, Dijon, France; 12 Institut Curie, Paris, France; 13 Centre Alexis Vautrin, Vandoeuvre les Nancy, Nancy, France; 14 Institut Curie, Saint-Cloud, France; 15 Centre Léon Berard, Lyon, France; 16 Centre Oscar Lambret, Lille, France; 17 Institut Jean Godinot, Reims, France; 18 Centre François Baclesse, Caen, France

## Abstract

**Importance:**

Breast cancer (BC) diagnosis and treatment expose patients to a 5-fold higher risk of depression compared with the general population, with an estimated prevalence of 10% to 25%. A depressive episode in patients with BC has implications for the tolerance of and adherence to treatment, impairing quality of life and reducing life expectancy.

**Objective:**

To identify and characterize distinct longitudinal patterns of depressive symptoms in patients with BC from diagnosis to 3 years after treatment.

**Design, Settings, and Participants:**

The CANTO-DEePRESS (Deeper in the Understanding and Prevention of Depression in Breast Cancer Patients) cohort study included women in the French multicenter CANTO (CANcer TOxicities) cohort study (conducted between March 20, 2012 and December 11, 2018), who were 18 years or older with invasive stage I to III BC and no previous BC treatment. The study aimed to characterize toxicities over a 5-year period following stage I to III primary BC treatment. Assessments of depressive symptoms were performed on a subset of patients with available data at diagnosis and at least 2 other time points. All data were extracted from the CANTO database on October 1, 2020.

**Main Outcomes and Measures:**

The primary outcome was the level of depressive symptoms at each assessment time point measured with the Hospital Anxiety and Depression Scale and depression subscale at BC diagnosis and at 3 to 6, 12, and 36 months after the end of treatment. The group-based trajectory modeling was used to identify trajectory groups, and multinomial logistic regression models were used to characterize the following factors associated with trajectory group affiliation: demographic, socioeconomic, clinical, lifestyle, and quality-of-life data.

**Results:**

A total of 4803 women (mean [SD] age, 56.2 [11.2] years; 2441 patients [50.8%] with stage I BC) were included in the study. Six trajectory groups that described the heterogeneity in the expression of depressive symptoms were identified: noncases with no expression of symptoms (n = 2634 [54.8%]), intermediate worsening (1076 [22.4%]), intermediate improvement (480 [10.0%]), remission (261 [5.4%]), delayed occurrence (200 [4.2%]), and stable depression (152 [3.2%]). HADS-D scores at diagnosis were consistently associated with the 5 depressive trajectory group affiliations, with an estimated higher probability per point increase of experiencing subthreshold or clinically significant depressive symptoms between diagnosis and the 3 years after the end of BC treatment. The higher probabilities ranged from 1.49 (95% CI, 1.43-1.54) for the intermediate worsening group to 10.53 (95% CI, 8.84-12.55) for the stable depression group. Trajectory groups with depressive symptoms differed from the noncases group without symptoms by demographic and clinical factors, such as having dependent children, lower household income, cancer stage, family history of BC, previous psychiatric hospitalizations, obesity, smoking status, higher levels of fatigue, and depression at diagnosis.

**Conclusions and Relevance:**

In this cohort study, nearly a third of patients with BC experienced temporary or lasting significant depressive symptoms during and after treatment. Improving early identification of women at risk of developing long-term or delayed depression is therefore critical to increase quality of life and overall survival. Subjected to validation, this study is an important first step toward personalized care of patients with BC at risk of depression.

## Key Points

**Question:**

Can distinct longitudinal patterns of depressive symptoms be characterized from diagnosis to 3 years after treatment for breast cancer?

**Findings:**

In this cohort study of 4803 women with breast cancer, 6 trajectory groups that described the heterogeneity in the expression of depressive symptoms were identified. The trajectory groups with depressive symptoms differed from the noncases group (without depressive symptoms) by household income, previous psychiatric hospitalizations, obesity, moderate to high levels of fatigue, and depression at diagnosis.

**Meaning:**

Findings of this study suggest that characterization of depressive trajectory groups after breast cancer diagnosis needs further validation but is a key step toward personalized management of patients at risk of depression, a common comorbidity in breast cancer associated with poorer prognosis.

## Teaser

This cohort study examines the progression of depressive symptoms and their associations with demographic, socioeconomic, clinical, lifestyle, and quality-of-life characteristics in women with invasive stage I to III breast cancer.

## Introduction

Breast cancer (BC) is the most common cancer worldwide and accounts for nearly 12% of new cases of cancer every year.^1^ In high-income countries, improvement in BC overall survival has been attributed to earlier diagnosis and access to more effective treatments, and the 5-year overall survival rate is approximately 85%.^2^ However, cross-sectional studies have shown a 5-fold increase in the risk for depression in women with BC compared with healthy women, with a prevalence rate of major depression ranging from 10% to 25%.^3,4^ The first year following the diagnosis is a critical phase when patients with BC are most likely to develop depressive symptoms.^5,6^ Such symptoms are associated with impaired quality of life, lower treatment adherence, prolonged hospitalization, and increased suicide risk.^7,8^ In addition, previous studies have found that depression at BC diagnosis is associated with worse overall survival.^9^

The group-based trajectory modeling (GBTM) approach is more reflective of the heterogeneity of patients’ adjustment over time than conventional studies, which focus on sample mean value changes and therefore do not account for the differences in the severity and course of symptoms.^10,11,12^ Previous trajectory studies suggested that, although most women (50%-80%) displayed minimal to no depressive symptoms over time (noncases group), 1% to 10% experienced substantial and persistent depressive symptoms from diagnosis and more than 2 to 5 years thereafter without remission (chronic group). Another 5% to 10% developed depressive symptoms with a delay (delayed group), and 10% to 20% exhibited symptoms at diagnosis and then progressively recovered, with a clear decrease in intensity within the first year (recovery group).^6,11,13,14,15^ However, estimating adjustment remains difficult and depends on the differences in screening tools and diagnostic cutoffs, number and timing of measures, sample size, duration of follow-up, and statistical approaches.^14,16^

With the exception of age, the factors associated with psychological trajectories (eg, educational level, marital status, socioeconomic characteristics, and social support) yielded varying conclusions; in particular, clinical parameters such as cancer stage and current and previous treatments.^5,6,17^ Younger women were more likely to develop and sustain depressive symptoms than older women.^6,15,17^ The implications of BC treatment for younger women were likely to be more disruptive for family and professional domains as well as fertility and sexuality because of the induced menopause.^18,19^

Although previous trajectory studies found different patterns of adjustment, they did not fully elucidate the interindividual variations associated with the development and evolution of depressive symptoms in patients with BC. Depression is an important and treatable complication of BC, and there is an unmet medical need for early identification of patients with depressive symptoms and those at risk of developing severe symptoms without remission to offer them appropriate, quick, and efficient care.

The CANTO-DEePRESS (Deeper in the Understanding and the Prevention of Depression in Breast Cancer Patients) study aims to identify and characterize distinct longitudinal patterns of depressive symptoms in patients with BC from diagnosis to 3 years after treatment. Ultimately, we aimed to develop a prognostic tool to better target and support patients with BC at a high risk for depression. The present article focuses on results obtained from the first ad hoc exploratory analyses performed on the national multicenter CANTO (Cancer Toxicities) cohort.^20^ The CANTO study aims to characterize long-term toxicities and their functional implications for BC survivors by collecting detailed information through clinical and paraclinical examinations, blood tests, and self-reported questionnaires.

## Methods

### Study Population

The CANTO-DEePRESS cohort study is based on a subset of data obtained from the CANTO cohort study, a French multicenter prospective observational study. The study was approved by the national regulatory authorities and local scientific committees (ID RCB: 2011-A01095-36). All included patients provided written informed consent. This substudy did not seek additional ethical approval. We followed the Strengthening the Reporting of Observational Studies in Epidemiology (STROBE) reporting guideline.

Full details about the CANTO cohort have been reported previously.^21^ Between March 20, 2012, and December 11, 2018, 12 012 patients were included from 26 centers in France. Eligible patients were women aged 18 years or older with a primary diagnosis of invasive stage I to III BC and who had not received any previous treatment (including surgery). Main exclusion criteria included metastatic or locally recurring BC, history of cancer within 5 years before inclusion, previous or current BC treatment, and a blood transfusion performed in the past 6 months before inclusion.

Patients completed assessment at diagnosis and then at 4 times after the end of primary treatment (surgery, chemotherapy, and/or radiotherapy): 3 to 6 months (T1), 12 months (T2), 36 months (T3), and 60 months (T4). Data collection and management were performed by the National French Cancer Centers Cooperative Group (UNICANCER).

### Measures

#### Primary Outcome

The level of depressive symptoms at each time point was considered as the primary outcome. It was measured with the depression subscale of the Hospital Anxiety and Depression Scale (HADS).^22^ Reliable in cancer settings, HADS is a widely used 14-item self-assessment scale.^23,24^ It consists of two 7-item subscales: one evaluates anxiety and one, depression (HADS-D).^25^ Each item is rated from 0 to 3, and total scores range from 0 to 21. Removal of somatic symptom measures from the questionnaire to prevent false-positive cases in patients with somatic symptoms has been found to make HADS especially relevant in detecting mental disorders in patients with cancer.^26^ The scores were interpreted as follows: 7 points or lower indicated noncases, 8 to 10 points indicated doubtful cases, and 11 points or higher indicated probable cases.^25^

### Covariates

Selected demographic, socioeconomic, clinical, lifestyle, and quality-of-life data were collected at diagnosis in patients’ case report form. These data included age, marital status, dependent children (0 to ≥1), level of education, working status, occupational category, household income, medical history of cancers (personal and familial), previous psychiatric hospitalizations and disorders, cancer stage and subtype, Charlson Comorbidity Index, menopausal status, body mass index (BMI), alcohol consumption, and smoking status. Pain, fatigue, insomnia, and satisfaction with body image were also measured at baseline using the European Organisation for Research and Treatment of Cancer Quality of Life Questionnaire (EORTC QLQC30-BR23); anxiety was measured using the HADS-A subscale; and level of physical activity was measured using the Global Physical Activity Questionnaire.^27,28,29^ All covariates were chosen a priori based on their availability and data from the literature on depression.

### Missing Data

Sensitivity analyses were also performed to assess patterns in HADS questionnaire completion over time. Patients missing 1 follow-up questionnaire were not different in terms of depressive-related covariates (eTables 2-4 in the Supplement). Given the random pattern of missing data, no imputation was performed.

### Identification of Trajectory Groups

Prevalence rates of patients with HADS-D scores that were categorized under noncases, doubtful cases, and probable cases were established at each assessment time point. To identify trajectories (the progression of longitudinal values of an outcome over time), we used the GBTM, which was developed by Nagin and colleagues.^30,31^ The GBTM is a statistical method to analyze trajectories and to delineate distinct subpopulations with similar trajectories. This approach considers latent groups with different unknown trajectory shapes and allows individual departures from the mean trajectory with a focus on change over time rather than on patterns of states.^32^ Trajectories were based on HADS-D scores over time and did not consider any other covariate in a fully exploratory approach. Two to 10 trajectories were successively tested, with linear, quadratic, and cubic functional forms. The optimal number of trajectories was chosen according to the bayesian information criterion, average group posterior probabilities (>70%), and odds of correct classification (≥5).^33^ The optimal functional form was defined by the χ^2^ test, with 2-sided *P* < .05 indicating statistical significance. The statistical modeling results were challenged for their clinical relevance (eTable 5 in the Supplement).

The GBTM analysis identified 7 trajectory groups of depressive symptoms with cubic functional form (eTable 5 in the Supplement). Two trajectory groups with a similar shape and consisting of patients with the lowest depressive mean scores over time were pooled according to clinical considerations. Therefore, the final classification comprised 6 trajectory groups (noncases, intermediate improvement, intermediate worsening, delayed occurrence, stable depression, and remission).

Once identified, the trajectories were characterized based on a multinomial logistic multivariable model, with a wide search for explanatory factors measured at baseline. Given the large number of trajectories and input covariates, the modeling process was implemented in several steps to enhance the convergence of estimates. Factors were first sorted into 2 classes according to their fixed (structural) or time-dependent (situational) nature, and 2 models were independently elaborated: (1) the epidemiological model included demographic, socioeconomic, and lifestyle candidate covariates, and (2) the clinical model included levels of anxiety, pain, fatigue, insomnia, satisfaction with body image, and depressive symptoms (eTables 7 and 8 in the Supplement). Depressive symptoms were entered into the clinical model using several coding schemes (continuous values and literature-based or data-driven classes). The continuous values turned out to be the most relevant coding for prognosis (convergence of model and discriminating power). Within each model, the covariates were screened with backward elimination procedure (5% threshold). Finally, an epidemioclinical model (hereafter referred to as the complete model) was created by gathering both epidemiological and clinical models, with an additional backward elimination procedure (5%).

### Statistical Analysis

#### Population Description

All of the data used were extracted from the database of the CANTO cohort that was dated October 1, 2020. Eligible patients had completed the HADS-D questionnaire at diagnosis and at least twice during the 36-month follow-up period (T1, T2, and/or T3). The comparison of the main demographic and clinical parameters at baseline between included and nonincluded patients showed no major evidence of loss of representativeness (eTable 1 in the Supplement).

#### Sensitivity Analyses

The GBTM approach was applied with and without dropout management to consider nonrandom attrition of patients (eTable 6 in the Supplement).^34^ The method was used with a dropout process depending on previous observations of depressive symptoms for level and trends. In the complete model, coding of covariates was investigated (pooling of modalities and/or variables) to optimize the modeling process (statistically and clinically); interactions and collinearities were systematically checked to deepen this process. Data analyses were performed using SAS, version 9.4 (SAS Institute, Inc).

## Results

A total of 6619 patients screened for eligibility, and 4803 (72.6%) were analyzed for trajectories (eFigure in the Supplement). The characteristics of the study population at the time of diagnosis are summarized in eTable 9 in the Supplement. This population had a mean (SD) age of 56.2 (11.2) years, and 70.8% (n = 3400) had partners or were married, 37.5% (n = 1803) had dependent children, and 52.6% (n = 2527) were employed. In addition, 50.8% of patients (n = 2441) had stage I BC, 29.0% (n = 1395) had an overweight BMI range, 19.2% (n = 920) had an obese BMI range, 10.3% (n = 493) reported pain, 23.8% (n = 1143) reported moderate to severe level of fatigue, 21.5% (n = 1031) were former smokers, 15.7% (n = 753) were current smokers, and 58.0% (n = 2786) engaged in sufficient physical activity.

### Prevalence of Depressive Symptoms Over Time

**Figure 1.**
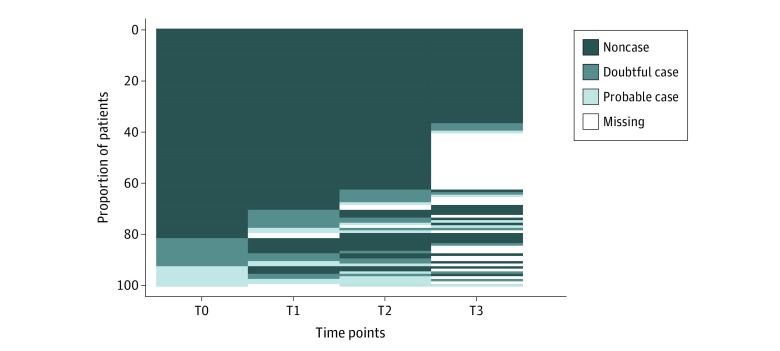
Prevalence of Depressive Symptoms at Each Time Point According to Hospital Anxiety and Depression Scale Clinical Thresholds Each line represents a patient’s Hospital Anxiety and Depression Scale score at each assessment time point: at diagnosis (T0) and at 3 to 6 months (T1), 12 months (T2), and 36 months (T3) after the end of primary treatment. Each score was categorized as follows: 7 or less indicates noncase; 8-10, doubtful case; and 11 or greater, probable case.

The proportion of patients in the doubtful cases category, with a HADS-D score of 8 to 10 points, tended to increase slightly over time, from 10.9% T0 to 13.9% T3, while the proportion of patients in the probable cases category, with a HADS-D score of 11 points or higher, remained relatively stable at approximately 7.0% (Figure 1). Over the 4 assessment time points, 16% percent of all patients had at least 1 HADS-D score that was categorized under probable cases.

**Figure 2.**
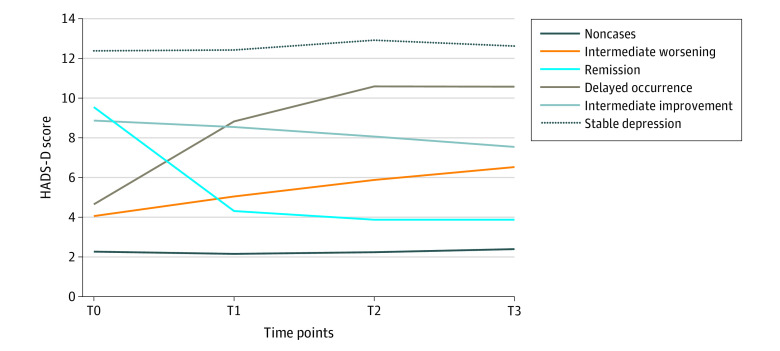
Identification of the Trajectory Groups of Depressive Symptoms in Patients With Breast Cancer HADS-D indicates Hospital Anxiety and Depression Scale, depression subscale; T0, at diagnosis; T1, 3 to 6 months; T2, 12 months; T3, 36 months.

Figure 2 shows the 6 trajectory groups, and the patient characteristics by trajectory group are provided in the Table. The noncases group comprised 2634 patients (54.8%) who had never reported any depressive symptoms. Conversely, the stable depression group comprised 152 patients (3.2%) with a consistently high level of depressive symptoms over time. The remission group (261 [5.4%]) was marked by a resolution of the depressive episode identified at diagnosis, and the delayed occurrence group (200 [4.2%]) was characterized by a later onset of depressive symptoms. The intermediate improvement (480 [10.0%]) and intermediate worsening (1076 [22.4%]) groups represented patients who maintained persistent depressive symptoms over time but showed either an improvement or a worsening of symptoms.

**Table.** OR Analysis of the Association of Depressive Symptom Covariates With Trajectory Group Affiliation

Covariate	Trajectory group vs noncases, OR (95% CI)^a^					Nullity test, *P* value
	Delayed occurrence	Intermediate improvement	Intermediate worsening	Remission	Stable depression	
With dependent children (reference: without dependent children)	1.27 (0.90-1.78)	0.86 (0.58-1.28)	0.99 (0.83-1.19)	1.38 (0.87-2.17)	0.69 (0.38-1.23)	.02
Household income (reference: <1500 per mo), €						
1500-3000	0.75 (0.48-1.17)	0.58 (0.34-0.99)^b^	0.80 (0.62-1.03)	0.67 (0.35-1.30)	0.55 (0.26-1.15)	.001
>3000	0.42 (0.26-0.69)^c^	0.40 (0.23-0.71)^c^	0.64 (0.50-0.83)^c^	0.58 (0.29-1.14)	0.24 (0.11-0.54)^c^	
Family history of BC (reference: no history)	1.15 (0.85-1.55)	1.32 (0.93-1.89)	1.23 (1.05-1.44)^b^	1.38 (0.91-2.09)	1.26 (0.74-2.15)	.002
Previous psychiatric hospitalizations (reference: no previous hospitalizations)	3.69 (1.8-7.55)^c^	3.17 (1.13-8.93)^b^	1.81 (1.09-3.00)^b^	2.48 (0.74-8.31)	4.59 (1.31-16.14)^b^	.02
Cancer stage (reference: stage I)						
Stage II	1.48 (1.07-2.04)^b^	1.28 (0.89-1.85)	1.02 (0.86-1.21)	1.19 (0.77-1.84)	1.53 (0.88-2.65)	.05
Stage III	1.39 (0.82-2.34)	2.68 (1.43-5.00)^c^	0.93 (0.70-1.25)	1.89 (0.90-4.00)	2.57 (1.04-6.36)^b^	
BMI range (reference: healthy BMI)						
Underweight	1.46 (0.50-4.26)	3.20 (0.97-10.55)	1.35 (0.79-2.30)	4.82 (1.29-17.95)^b^	3.35 (0.45-25.02)	<.001
Overweight	1.84 (1.28-2.65)^c^	1.64 (1.08-2.50)^b^	1.60 (1.33-1.93)^c^	1.17 (0.71-1.92)	1.39 (0.74-2.60)	
Obesity	2.29 (1.53-3.42)^c^	2.75 (1.71-4.40)^c^	1.69 (1.36-2.11)^c^	1.88 (1.07-3.32)^b^	2.45 (1.20-5.00)^c^	
Smoking status (reference: nonsmoking)						
Former	0.97 (0.66-1.43)	1.07 (0.68-1.67)	1.02 (0.83-1.24)	1.35 (0.81-2.25)	1.6 (0.83-3.09)	.004
Current	1.48 (0.98-2.24)	1.97 (1.22-3.18)^c^	1.62 (1.30-2.03)^c^	1.75 (0.99-3.09)	4.03 (2.07-7.86)^c^	
Fatigue level ≥ 40(reference: score <40)	2.46 (1.75-3.45)^c^	2.49 (1.71-3.63)^c^	2 (1.64-2.44)^c^	1.49 (0.96-2.32)	4.02 (2.31-7.00)^c^	<.001
HADS-D score per point increase	1.53 (1.42-1.63)^c^	6.27 (5.39-7.31)^c^	1.49 (1.43-1.54)^c^	7.7 (6.55-9.07)^c^	10.53 (8.84-12.55)^c^	<.001

Abbreviations: BC, breast cancer; BMI, body mass index (calculated as weight in kilograms divided by height in meters squared); HADS-D, Hospital Anxiety and Depression Scale, depression subscale; OR, odds ratio.

To convert euros to US dollars, multiply by 1.12.

^a^ ORs were statistically significant at 5% (based on joint tests).

^b^ *P* < .05.

^c^ *P* < .01.

### Factors Associated With Membership in Trajectory Groups

**Figure 3.**
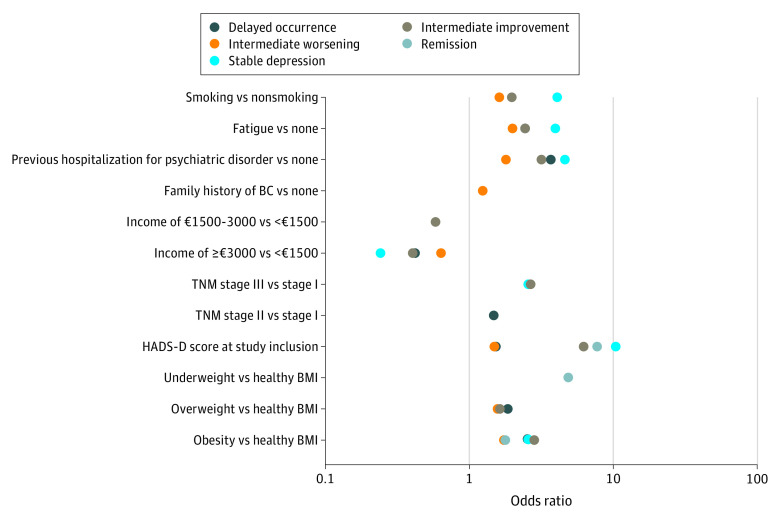
Association of Depressive Symptom Covariates With Trajectory Group Affiliation in the Prognostic Model Only odds ratios (ORs) that were significant at 5% are represented. All OR estimates and CIs are provided in the Table; CIs are not represented in this figure. BC indicates breast cancer; BMI, body mass index (calculated as weight in kilograms divided by height in meters squared); and HADS-D, Hospital Anxiety and Depression Scale, depression subscale.

The multinomial logistic regression model showed significant associations between both structural and situational factors and patient affiliation with the 5 depressive symptom trajectory groups: intermediate improvement, intermediate worsening, delayed occurrence, remission, and stable depression) (Table). Specifically, demographic (dependent children and household income) and clinical (cancer stage and BMI) characteristics, medical history (family history of BC and previous psychiatric hospitalization), lifestyle habits (obesity and smoking status), and symptoms (depression and fatigue levels) at diagnosis were associated (albeit at different degrees) with the duration and severity of depression experienced by patients in the trajectory groups compared with those in the noncases group (Figure 3).

HADS-D scores at diagnosis were consistently associated with the 5 depressive trajectory group affiliations, with an estimated higher probability per point increase of experiencing subthreshold or clinically significant depressive symptoms between diagnosis and the 3 years after the end of BC treatment. The higher probabilities ranged from 1.49 (95% CI, 1.43-1.54) for the intermediate worsening group to 10.53 (95% CI, 8.84-12.55) for the stable depression group. In addition, as shown in the Table, we observed a gradual association of lower household income, previous psychiatric hospitalizations, obesity, moderate to high levels of fatigue, and depression at diagnosis with the stable depression, delayed occurrence, and intermediate worsening trajectory groups.

## Discussion

The CANTO DEePRESS cohort study aimed to identify and characterize distinct longitudinal patterns of depressive symptoms between the time of diagnosis and 3 years post BC treatment. Six distinct trajectory groups emerged from our analysis, 4 of which were consistent with results of previous research in patients with BC.^6,11,13,14,15^ The findings confirmed the 4 commonly observed patterns of the evolution of depressive symptoms in patients within BC: most patients (54.8%) did not exhibit symptoms (noncases), 3.2% of patients consistently reported substantial levels of depressive symptoms (stable depression trajectory), 5.4% of patients recovered from an initial depressive period (remission trajectory), and 4.2% of patients developed time-delayed depressive symptoms (delayed occurrence trajectory). The modeling also identified 2 other emerging trajectory groups, intermediate improvement and intermediate worsening, which are not well described in the literature but provided new insights into the existence of less contrasted longitudinal patterns. The intermediate improvement (10.0%) and the intermediate worsening (22.4%) groups accounted for more than a third of the patients.

Avis et al^6^ have described a similar proportion of patients (29%) with borderline levels of depressive symptoms among BC survivors during the 2 years after the diagnosis. The present study suggests a different dynamic within these borderline groups. From a clinical point of view, 1 of 5 patients with the intermediate worsening trajectory displayed a progressive worsening of their emotional state, although the diagnosis of depression could not be fully confirmed over time. This result highlights the importance of understanding the mechanisms behind the evolution of depressive symptoms in particular intermediate groups. Furthermore, several risk factors surfaced when comparing the noncases and the trajectory groups: lower household income, previous psychiatric hospitalizations, obesity, moderate to high levels of fatigue, and depression at diagnosis.

**Figure 4.**
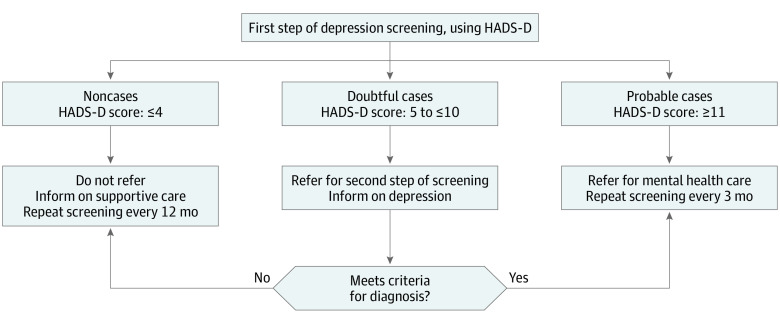
Proposal of Care Model Process for Depression Screening in Patients With Breast Cancer at Diagnosis HADS-D indicates Hospital Anxiety and Depression Scale, depression subscale.

These results suggest that special attention should be paid to looking for these factors over time starting from BC diagnosis, and affected patients could be identified early with an appropriate protocol and referred to supportive care. The HADS-D score appeared to be one of the most discriminating factors, which supports the recommendations of using the questionnaire as an essential first step in early screening for depression.^5,35^ As such, a score of 4 points or lower would indicate a low risk of long-term depression that does not require special intervention, whereas a score of 11 points or higher would indicate a high risk of long-lasting depression that justifies a referral for mental health care. However, our study also showed the limitations of interpreting HADS scores between 5 and 10 at diagnosis and accurately anticipating borderline longitudinal patterns. As suggested in a previous meta-analysis,^36^ in such cases, referral to a second step of depression screening (ie, further assessment through an open results discussion with patients, semistructured or fully structured diagnostic interview, or completion of other self-report questionnaires) and follow-up consultations could be recommended to patients (Figure 4 shows the Care Model Process).

In addition, the results of this study stress the need for new investigations to better identify the most sensitive HADS-D questionnaire items and clinical thresholds for differentiating the various conditions of the depressive spectrum disorders.^23,24^ Indeed, it is essential to grasp the complex characteristics defining the intermediate trajectory patterns to provide the best supportive care possible to all patients.^35^ If considering new cutoffs for HADS scores or integrating different screening tools, a stepped-care pathway would be useful in accurately detecting more depressive symptoms. Such practices are presently restricted by the risk of overestimating or underestimating depressive symptoms and by the lack of systematic screening in routine care.^7,37,38,39^

We believe the CANTO-DEePRESS study offers a unique opportunity to make further progress in this area by integrating both dynamic and heterogeneous dimensions of depressive symptoms over an extended period. This large study allows us to consider symptomatological issues and the interplay between several potential covariates at the same time as well as characterization of the various conditions. In this way, the findings provide an avenue to apprehend the conditions of occurrence and disappearance of depressive symptoms during and after BC treatment by subsequently integrating additional patient characteristics and time-dependent factors into the GBTM.

### Limitations

This study has several limitations. Despite a large sample size and extended follow-up period, which most likely highlighted the intermediate trajectory groups, biases based on single-nation design and missing data may prevent generalization to all patients with BC. This bias was assessed as far as possible through sensitivity analyses. Furthermore, the internal validity of the complete model may have been affected by unmeasured cofounding factors and the lack of a cross-check procedure, including a professional evaluation to formally diagnose depression.^7^ However, the integration of new risk factors may partly balance these limitations. Data on previous psychiatric hospitalization, for example, were missing in most trials but emerged as one of the key factors associated with trajectory group affiliations in the final model. It has previously been shown as a factor in future morbidity among patients with BC.^19^ This result supports the interest of the GBTM used in this study, which will be further investigated and validated during the development of a prognostic score and will be enriched by time-dependent factors.

## Conclusions

This CANTO DEePRESS study showed that, behind an apparent stability in the prevalence of depression from diagnosis to 3 years after BC treatment, it was possible to identify a minimum of 6 trajectory groups (including 1 without depressive symptoms). The characterization of patient profiles across the different depressive trajectory groups (compared with the group without depressive symptoms) highlighted several key factors that were associated with depressive symptoms in patients with BC: lower household income, previous psychiatric hospitalizations, obesity, moderate to high levels of fatigue, and depression at diagnosis. These findings are promising for a forthcoming validation of an enriched modeling study, which aims to better guide the management and care of patients at risk for depression at an early stage.

## Notes

Supplement. **eTable 1.** Comparison of Baseline Demographic and Clinical Characteristics Among Patients Included or Not in the CANTO-DEePRESS Study **eTable 2.** Patterns of Missingness in HADS Questionnaire Completion Over Time (T0-T1-T2-T3) (n = 4803) **eTable 3.** Missingness in HADS Questionnaire Completion at T3 According to Literature-based Trajectories **eTable 4.** Additional Psychological Information Regarding Missingness in the Completion of the HADS Questionnaire at T3 **eFigure.** Flowchart of CANTO-DEePRESS Study Population **eTable 5.** Fit Indices from GBTM Approach to Identify Depressive Symptom Trajectory-Groups **eTable 6.** Fit Indices from GBTM Approach to Identify Depressive Symptom Trajectory-Groups—Sensitivity Analysis With Dropout Management **eTable 7.** Epidemiological Model Outcomes **eTable 8.** Clinical Model Outcomes **eTable 9.** Description of Baseline Patients’ Characteristics Based on Trajectory Affiliation
