# Supplementary material for: Characterization of Depressive Symptoms Trajectories After Breast Cancer Diagnosis in Women in France
Source: JAMA Netw Open. 2022 Apr 14;5(4):e225118. doi: 10.1001/jamanetworkopen.2022.5118 (PMC9011125; doi:10.1001/jamanetworkopen.2022.5118)
Supplement: Supplement. — eTable 1. Comparison of Baseline Demographic and Clinical Characteristics Among Patients Included or Not in the CANTO-DEePRESS Study eTable 2. Patterns of Missingness in HADS Questionnaire Completion Over Time (T0-T1-T2-T3) (n = 4803) eTable 3. Missingness in HADS Questionnaire Completion at T3 According to Literature-based Trajectories eTable 4. Additional Psychological Information Regarding Missingness in the Completion of the HADS Questionnaire at T3 eFigure. Flowchart of CANTO-DEePRESS Study Population eTable 5. Fit Indices from GBTM Approach to Identify Depressive Symptom Trajectory-Groups eTable 6. Fit Indices from GBTM Approach to Identify Depressive Symptom Trajectory-Groups—Sensitivity Analysis With Dropout Management eTable 7. Epidemiological Model Outcomes eTable 8. Clinical Model Outcomes eTable 9. Description of Baseline Patients’ Characteristics Based on Trajectory Affiliation [file jamanetwopen-e225118-s001.pdf]

Supplementary Online Content

Charles C, Bardet A, Larive A, et al. Characterization of depressive symptoms trajectories after breast cancer diagnosis in women in France. *JAMA Netw Open*. 2022;5(4):e225118. doi:10.1001/jamanetworkopen.2022.5118

eTable 1. Comparison of Baseline Demographic and Clinical Characteristics Among Patients Included or Not in the CANTO-DEePRESS Study

eTable 2. Patterns of Missingness in HADS Questionnaire Completion Over Time (T0-T1-T2-T3) (n=4803)

eTable 3. Missingness in HADS Questionnaire Completion at T3 According to Literature-based Trajectories

eTable 4. Additional Psychological Information Regarding Missingness in the Completion of the HADS Questionnaire at T3

eFigure. Flowchart of CANTO-DEePRESS Study Population

eTable 5. Fit Indices from GBTM Approach to Identify Depressive Symptom Trajectory-Groups

eTable 6. Fit Indices from GBTM Approach to Identify Depressive Symptom Trajectory-Groups—Sensitivity Analysis With Dropout Management

eTable 7. Epidemiological Model Outcomes

eTable 8. Clinical Model Outcomes

eTable 9. Description of Baseline Patients' Characteristics Based on Trajectory Affiliation

This supplementary material has been provided by the authors to give readers additional information about their work.

© 2022 Charles C et al. *JAMA Network Open.*

eTable 1. Comparison of Baseline Demographic and Clinical Characteristics Among Patients Included or Not in the CANTO-DEePRESS Study

	**Patients included** (n=4803)	**Patients not included** (n=1816)	**All** (n=6619)
**Characteristics**	n (%)	n (%)	n (%)
**Age** (years)			
<40	323 (6.7)	129 (7.1)	452 (6.8)
[40-75]	4314 (89.8)	1578 (86.9)	5892 (89.0)
>75	166 (3.5)	109 (6.0)	275 (4.2)
**Working status**			
Active	2527 (52.6)	707 (38.9)	3234 (48.9)
Inactive	2218 (46.2)	734 (40.4)	2952 (44.6)
Missing	58 (1.2)	375 (20.6)	433 (6.5)
**Monthly household income**			
<1500	620 (12.9)	299 (16.5)	919 (13.9)
[1500-3000]	1754 (36.5)	530 (29.2)	2284 (34.5)
>3000	1967 (41.0)	442 (24.3)	2409 (36.4)
Missing	462 (9.6)	545 (30.0)	1007 (15.2)
**TNM stage**			
Stage I	2441 (50.8)	804 (44.3)	3245 (49.0)
Stage II	1907 (39.7)	793 (43.7)	2700 (40.8)
Stage III	452 (9.4)	189 (10.4)	641 (9.7)
Missing	3 (0.1)	30 (1.7)	33 (0.5)
**Charlson comorbidity index**			
0	3567 (74.3)	1269 (69.9)	4836 (73.1)
1	458 (9.5)	218 (12.0)	676 (10.2)
≥2	434 (9.0)	152 (8.4)	586 (8.9)
Missing	344 (7.2)	177 (9.7)	521 (7.9)

© 2022 Charles C et al. *JAMA Network Open.*

	Patients included (n=4803)	Patients not included (n=1816)	All (n=6619)
**Characteristics**	n (%)	n (%)	n (%)
**Pain**			
<40	4294 (89.4)	1206 (66.4)	5500 (83.1)
≥40	493 (10.3)	215 (11.8)	708 (10.7)
Missing	16 (0.3)	395 (21.8)	411 (6.2)
**Fatigue**			
<40	3649 (76.0)	1010 (55.6)	4659 (70.4)
≥40	1143 (23.8)	414 (22.8)	1557 (23.5)
Missing	11 (0.2)	392 (21.6)	403 (6.1)
**Former psychiatric hospitalization**			
Yes	131 (2.7)	60 (3.3)	191 (2.9)
No	4578 (95.3)	1692 (93.2)	6270 (94.7)
Missing	94 (2.0)	64 (3.5)	158 (2.4)
**Depression (HADS-D)**			
[0-7]	3946 (82.2)	1049 (57.8)	4995 (75.5)
[8-10]	523 (10.9)	173 (9.5)	696 (10.5)
≥11	334 (7.0)	97 (5.3)	431 (6.5)
Missing	0 (0.0)	497 (27.4)	497 (7.5)
**Anxiety (HADS-A)**			
[0-7]	1868 (38.9)	516 (28.4)	2384 (36.0)
[8-10]	1279 (26.6)	371 (20.4)	1650 (24.9)
≥11	1651 (34.4)	436 (24.0)	2087 (31.5)
Missing	5 (0.1)	493 (27.1)	498 (7.5)

Note: Percents were calculated using the total number of patients indicated at the top of each column.

© 2022 Charles C et al. *JAMA Network Open.*

eTable 2. Patterns of Missingness in HADS Questionnaire Completion Over Time (T0-T1-T2-T3) (n=4803)

Pattern of missingness[a]	Proportion of patients n (%)	Pattern of missingness	Proportion of patients n (%)
0-.-0-0	114 (2.37)	1-.-0-0	4 (0.08)
0-.-0-1	7 (0.15)	1-.-0-1	1 (0.02)
0-.-1-0	7 (0.15)	1-.-1-1	1 (0.02)
0-.-1-1	3 (0.06)	1-0-.-0	7 (0.15)
0-0-.-0	146 (3.04)	1-0-.-1	3 (0.06)
0-0-.-1	17 (0.35)	1-0-0-.	67 (1.39)
0-0-0-.	1361 (28.34)	1-0-0-0	97 (2.02)
0-0-0-0	2407 (50.11)	1-0-0-1	13 (0.27)
0-0-0-1	88 (1.83)	1-0-1-.	14 (0.29)
0-0-1-.	57 (1.19)	1-0-1-0	14 (0.29)
0-0-1-0	58 (1.21)	1-0-1-1	6 (0.12)
0-0-1-1	23 (0.48)	1-1-.-0	3 (0.06)
0-1-.-0	10 (0.21)	1-1-.-1	1 (0.02)
0-1-.-1	3 (0.06)	1-1-0-.	14 (0.29)
0-1-0-.	36 (0.75)	1-1-0-0	14 (0.29)
0-1-0-0	54 (1.12)	1-1-0-1	8 (0.17)
0-1-0-1	12 (0.25)	1-1-1-.	27 (0.56)
0-1-1-.	31 (0.65)	1-1-1-0	10 (0.21)
0-1-1-0	18 (0.37)	1-1-1-1	30 (0.62)
0-1-1-1	17 (0.35)	1-.-0-0	4 (0.08)
		1-.-0-1	1 (0.02)

[a] 0: HADS< 11; 1: HADS≥ 11

Comments: eTable 2 shows patterns of missingness. When missingness was temporary and data reintroduced later during the study (missingness at a time other than T3), we considered that there was no major evidence for non-randomness of missingness.

© 2022 Charles C et al. *JAMA Network Open.*

eTable 3. Missingness in HADS Questionnaire Completion at T3 According to Literature-based Trajectories

	**Proportion of patients n (%)**	**Proportion of patients with no missing data n (%)**
Total	4803	2869 (59.7%)[a]
Non-cases trajectory	4028 (83.9%)[a]	2407 (83.9%)[b]
Delayed trajectory	441 (9.2%)[a]	270 (9.4%)[b]
Chronic trajectory	104 (2.2%)[a]	57 (2.0%)[b]
Recovery trajectory	230 (4.8%)[a]	135 (4.7%)[b]

Note: Percents were calculated based on a) total study population (n=4803) or b) proportion of patients with no missing data.

Comments: Considering literature-based trajectories in n=2869 patients (59.7%) with fully completed HADS-D questionnaires, there was no major evidence for non-randomness of missingness.

© 2022 Charles C et al. *JAMA Network Open.*

eTable 4. Additional Psychological Information Regarding Missingness in the Completion of the HADS Questionnaire at T3

	**Population with missing HADS questionnaire at T3 (n=1607)**	**Population with no missing HADS questionnaire at T3 (n=3196)**
**Variables (since last study visit)**	n (%)	n (%)
**Psychiatric hospitalizations**		
No	1602 (99.7)	3174 (99.3)
Yes	5 (0.3)	22 (0.7)
**Psychiatric consultations**		
No	1578 (98.2)	3031 (94.8)
Yes	29 (1.8)	165 (5.2)
**Psychological consultations**		
No	1540 (95.8)	2972 (93.0)
Yes	67 (4.2)	224 (7.0)

Comments: Complementary variables were analyzed in order to better characterize missing data and to decide whether imputation was appropriate or not. There was no meaningful difference in psychological events (hospitalizations or consultations) when comparing patients with and without full data at T3.

Conclusion about missing data: Missingness in HADS questionnaire completion was greater at T3. However, there was no major evidence for bias and therefore, no imputation was done.

© 2022 Charles C et al. *JAMA Network Open.*

eFigure. Flowchart of CANTO-DEePRESS Study Population

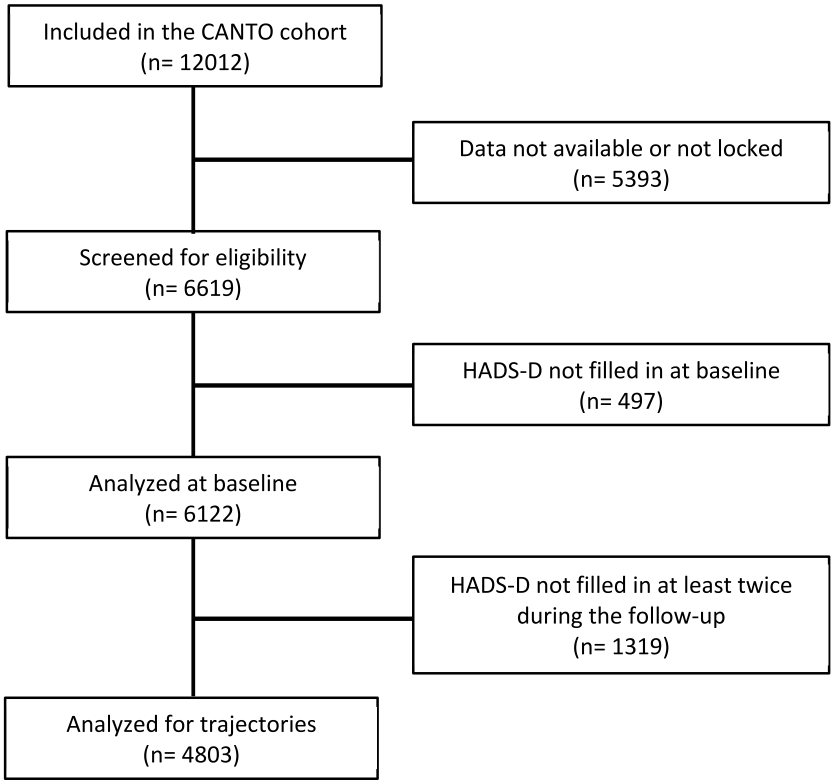

© 2022 Charles C et al. *JAMA Network Open.*

eTable 5. Fit Indices from GBTM Approach to Identify Depressive Symptom Trajectory-Groups

Model	BIC	χ2	Group 1			Group 2			Group 3			Group 4			Group 5			Group 6			Group 7			Group 8			Group 9			Group 10		
			n	OCC	AvPP	n	OCC	AvPP	n	OCC	AvPP	n	OCC	AvPP	n	OCC	AvPP	n	OCC	AvPP	n	OCC	AvPP	n	OCC	AvPP	n	OCC	AvPP	n	OCC	AvPP
2 trajectories with linear polynomial functional form	-42834,9977	P<.001	3375	9,1	95,5%	1428	23,4	91,0%	.	.	,	.	.	,	.	.	,	.	,	,	,	,	,	,	,	,	,	,	,	,	,	,
3 trajectories with linear polynomial functional form	-42302,4271	P<.001	2614	9,1	91,3%	1791	10,0	85,9%	398	75,4	87,8%	.	.	,	.	.	,	.	,	,	,	,	,	,	,	,	,	,	,	,	,	,
4 trajectories with linear polynomial functional form	-42135,3325	P<.001	1507	9,9	82,0%	2088	4,9	78,1%	1026	19,5	84,7%	182	187,9	88,8%	.	.	,	.	,	,	,	,	,	,	,	,	,	,	,	,	,	,
5 trajectories with linear polynomial functional form	-42063,6995	P<.001	1323	10,9	81,0%	2158	4,6	77,2%	411	16,8	67,6%	710	15,9	72,6%	201	167,3	88,3%	.	,	,	,	,	,	,	,	,	,	,	,	,	,	,
6 trajectories with linear polynomial functional form	-42036,5743	P<.001	219	40,2	72,0%	2093	5,7	80,0%	1468	5,5	69,9%	273	25,0	67,9%	575	19,2	72,2%	175	167,4	86,4%	,	,	,	,	,	,	,	,	,	,	,	,
7 trajectories with linear polynomial functional form	-42013,5929	P<.001	267	34,0	70,1%	2146	5,4	79,1%	1114	5,0	59,8%	380	13,9	62,2%	321	25,8	65,9%	452	16,5	63,6%	123	207,8	84,4%	,	,	,	,	,	,	,	,	,
8 trajectories with linear polynomial functional form	-42013,6447	P<.001	157	58,7	76,2%	1895	5,6	76,2%	1249	4,3	58,7%	306	16,7	58,2%	567	10,9	61,2%	63	87,4	65,4%	452	19,7	65,2%	114	239,7	85,6%	,	,	,	,	,	,
9 trajectories with linear polynomial functional form	-42028,2795	P<.001	282	28,9	61,2%	0	.	,	1562	3,2	45,3%	1457	3,9	56,2%	567	10,9	61,2%	63	87,4	65,4%	452	19,7	65,2%	306	16,7	58,2%	114	239,7	85,6%	,	,	,
10 trajectories with linear polynomial functional form	-42008,8416	P<.001	229	40,0	72,2%	1231	4,6	58,8%	2053	5,6	78,6%	123	24,5	56,1%	166	27,0	64,5%	737	13,1	66,6%	55	94,7	65,6%	26	295,1	71,5%	159	86,5	74,1%	24	879	83,8%
2 trajectories with quadratic polynomial functional form	-42836,2747	P<.001	3378	9,1	95,5%	1425	23,4	91,0%	.	.	,	.	.	,	.	.	,	.	.	,	.	.	,	.	.	,	.	.	,	,	,	,
3 trajectories with quadratic polynomial functional form	-42307,0929	P<.001	2592	9,4	91,5%	1806	9,7	85,6%	405	74,4	87,9%	.	.	,	.	.	,	.	.	,	.	.	,	.	.	,	.	.	,	,	,	,
4 trajectories with quadratic polynomial functional form	-42143,2603	P<.001	1526	10,0	82,6%	2067	5,0	78,3%	1030	18,7	83,9%	180	190,3	88,9%	.	.	,	.	.	,	.	.	,	.	.	,	.	.	,	,	,	,
5 trajectories with quadratic polynomial functional form	-42011,3618	P<.001	1386	11,0	82,2%	2101	5,0	78,2%	675	18,5	75,8%	425	24,3	72,6%	216	156,9	88,3%	.	.	,	.	.	,	.	.	,	.	.	,	,	,	,
6 trajectories with quadratic polynomial functional form	-41960,5454	P<.001	602	19,9	77,1%	2299	5,0	80,2%	888	10,1	70,1%	464	31,1	77,4%	427	24,0	71,8%	123	225,6	85,9%	.	.	,	.	.	,	.	.	,	,	,	,
7 trajectories with quadratic polynomial functional form	-41927,3299	P<.001	366	30,9	77,1%	2270	5,4	80,6%	1105	8,2	71,2%	154	62,0	72,2%	507	23,4	73,1%	253	34,0	69,0%	148	192,6	85,9%	.	.	,	.	.	,	,	,	,
8 trajectories with quadratic polynomial functional form	-41916,9536	P<.001	381	30,7	77,8%	2284	5,3	80,1%	1059	7,8	67,7%	75	66,1	68,8%	162	60,6	71,6%	223	39,9	69,6%	472	25,0	73,2%	147	183,7	84,9%	.	.	,	,	,	,
9 trajectories with quadratic polynomial functional form	-41911,2005	P<.001	357	31,9	76,7%	1053	7,3	66,4%	2214	5,5	80,0%	524	19,8	70,1%	82	60,0	67,3%	202	43,7	69,5%	169	60,4	72,5%	177	101,0	79,3%	25	988,4	86,7%	,	,	,
10 trajectories with quadratic polynomial functional form	-41920,4054	P<.001	153	58,6	74,2%	1507	5,6	68,9%	1194	4,1	56,8%	763	8,8	61,1%	88	57,5	67,3%	122	77,0	71,2%	306	23,9	65,2%	497	23,7	72,6%	29	391,5	71,9%	144	190	85,1%
2 trajectories with cubic polynomial functional form	-42844,2529	0,2	3382	9,0	95,4%	1421	23,6	91,1%	.	.	,	.	.	,	.	.	,	.	.	,	.	.	,	.	.	,	.	.	,	,	,	,
3 trajectories with cubic polynomial functional form	-42319,6722	0,2	2586	9,3	91,4%	1811	9,6	85,4%	406	76,6	88,4%	.	.	,	.	.	,	.	.	,	.	.	,	.	.	,	.	.	,	,	,	,
4 trajectories with cubic polynomial functional form	-42154,9325	0,004	1601	9,8	83,3%	2040	5,1	78,3%	981	20,0	84,3%	181	175,5	87,8%	.	.	,	.	.	,	.	.	,	.	.	,	.	.	,	,	,	,
5 trajectories with cubic polynomial functional form	-41971,3883	P<.001	1428	11,0	82,6%	2015	5,3	78,1%	866	19,0	80,6%	277	45,1	76,8%	217	155,6	88,2%	.	.	,	.	.	,	.	.	,	.	.	,	,	,	,
6 trajectories with cubic polynomial functional form	-41907,9131	P<.001	576	20,4	76,0%	2242	5,3	80,5%	946	9,9	71,4%	555	27,8	78,5%	343	37,7	76,8%	141	233,5	88,1%	.	.	,	.	.	,	.	.	,	,	,	,
7 trajectories with cubic polynomial functional form	-41855,7866	P<.001	437	26,1	76,1%	2197	5,7	80,8%	200	54,7	73,0%	1076	9,3	72,9%	480	25,3	74,1%	261	40,9	72,7%	152	209,2	87,1%	.	.	,	.	.	,	,	,	,
8 trajectories with cubic polynomial functional form	-41825,1996	P<.001	494	23,8	75,6%	116	56,6	65,0%	2141	5,9	81,1%	1017	9,7	72,0%	294	37,2	72,9%	184	54,9	70,4%	414	28,9	72,9%	143	212,1	86,4%	.	.	,	,	,	,
9 trajectories with cubic polynomial functional form	-41817,2622	P<.001	365	32,9	78,0%	2146	5,8	80,1%	1053	8,0	68,7%	141	48,1	65,1%	142	57,2	69,7%	77	129,4	72,1%	482	25,7	72,7%	271	42,9	74,5%	126	244,2	86,6%	,	,	,
10 trajectories with cubic polynomial functional form	-41778,8397	P<.001	375	32,9	78,4%	948	8,9	67,3%	2136	5,9	80,0%	509	25,2	74,2%	106	56,3	69,3%	64	141,3	71,9%	143	81,0	73,7%	331	31,3	72,2%	37	544,6	84,0%	154	241	88,8%

BIC: Bayesian Information Criterion based on the number of observations (to maximize) / X^2: P value for Chi-square test of nullity of higher degree terms / n: number of patients

Comments: The predefined statistical criteria for choice of optimal trajectory-groups classification are: Chi-square test for functional form with p value <0,05, BIC to maximize, Average group Posterior Probabilities (AvPP) >70.0%, and Odds of Correct Classification (OCC) ≥5.

Conclusion: There is a requirement of cubic functional form from a classification into 4 groups, the optimal average posterior probability is for 7 trajectories or less**, and BIC criteria is in favor of high numbers of trajectories. The other statistical criteria are not discriminant.**

© 2022 Charles C et al. *JAMA Network Open.*

eTable 6. Fit Indices from GBTM Approach to Identify Depressive Symptom Trajectory-Groups—Sensitivity Analysis With Dropout Management

Model	BIC	χ2	Group 1			Group 2			Group 3			Group 4			Group 5			Group 6			Group 7			Group 8			Group 9			Group 10		
			n	OCC	AvPP	n	OCC	AvPP	n	OCC	AvPP	n	OCC	AvPP	n	OCC	AvPP	n	OCC	AvPP	n	OCC	AvPP	n	OCC	AvPP	n	OCC	AvPP	n	OCC	AvPP
2 trajectories with linear polynomial functional form	-47124,68820	P<.001	3383	8,9	95,4%	1420	24,0	91,2%	.	.	.	.	.	.	.	.	.	.	.	.	.	.	.	.	.	.	.	.	.	.	.	.
3 trajectories with linear polynomial functional form	-46606,46310	P<.001	2611	9,3	91,5%	1806	9,8	85,6%	386	82,6	88,7%	.	.	.	.	.	.	.	.	.	.	.	.	.	.	.	.	.	.	.	.	.
4 trajectories with linear polynomial functional form	-46453,73090	P<.001	1500	9,8	81,9%	2091	4,9	78,2%	1027	19,6	84,8%	185	176,9	88,2%	.	.	.	.	.	.	.	.	.	.	.	.	.	.	.	.	.	.
5 trajectories with linear polynomial functional form	-46349,8958	P<.001	1327	11,2	81,7%	2153	5,0	78,6%	578	18,5	72,9%	520	21,7	73,8%	225	159,7	88,9%	.	.	.	.	.	.	.	.	.	.	.	.	.	.	.
6 trajectories with linear polynomial functional form	-46308,01290	P<.001	730	17,4	78,8%	2318	4,8	79,5%	703	12,7	69,8%	421	32,9	75,7%	524	19,8	71,8%	107	298,5	88,1%	.	.	.	.	.	.	.	.	.	.	.	.
7 trajectories with linear polynomial functional form	-46293,88060	P<.001	383	29,3	76,7%	2352	5,3	81,0%	440	13,7	63,4%	813	10,9	68,6%	213	47,3	70,0%	461	25,2	73,1%	141	242,8	88,2%	.	.	.	.	.	.	.	.	.
8 trajectories with linear polynomial functional form	-46297,74800	P<.001	388	29,7	77,7%	2351	5,3	80,8%	485	13,5	64,4%	33	295,3	72,4%	732	12,0	68,6%	162	61,0	69,5%	519	20,6	70,8%	133	256,3	88,2%	.	.	.	.	.	.
9 trajectories with linear polynomial functional form	-46303,66610	P<.001	195	47,4	73,6%	1876	5,8	76,3%	217	32,3	61,0%	1099	5,9	64,9%	631	13,0	65,5%	102	85,4	66,7%	313	26,0	65,6%	270	37,9	70,5%	100	265,9	85,2%	.	.	.
10 trajectories with linear polynomial functional form	-46332,93560	P<.001	294	29,4	63,4%	0	.	.	1564	3,5	49,5%	1308	5,0	61,3%	221	31,6	60,6%	631	13,0	65,5%	102	85,4	66,7%	313	26,0	65,6%	270	37,9	70,5%	100	265,9	85,2%
2 trajectories with quadratic polynomial functional form	-47125,37030	P<.001	3386	8,9	95,4%	1417	24,1	91,2%	.	.	.	.	.	.	.	.	.	.	.	.	.	.	.	.	.	.	.	.	.	.	.	.
3 trajectories with quadratic polynomial functional form	-46609,91480	P<.001	2601	9,5	91,6%	1811	9,7	85,5%	391	82,7	88,9%	.	.	.	.	.	.	.	.	.	.	.	.	.	.	.	.	.	.	.	.	.
4 trajectories with quadratic polynomial functional form	-46459,85070	P<.001	1520	10,1	82,7%	2077	5,0	78,4%	1022	19,0	84,0%	184	176,1	88,2%	.	.	.	.	.	.	.	.	.	.	.	.	.	.	.	.	.	.
5 trajectories with quadratic polynomial functional form	-46236,82360	P<.001	1316	11,4	81,8%	2094	5,2	78,7%	687	21,7	78,9%	463	27,3	75,9%	243	153,0	89,2%	.	.	.	.	.	.	.	.	.	.	.	.	.	.	.
6 trajectories with quadratic polynomial functional form	-46171,42090	P<.001	636	18,5	76,2%	2208	5,4	80,5%	784	12,7	72,4%	477	31,5	77,5%	547	24,2	75,9%	151	215,8	88,1%	.	.	.	.	.	.	.	.	.	.	.	.
7 trajectories with quadratic polynomial functional form	-46122,06970	P<.001	336	32,3	74,7%	2171	6,2	82,1%	452	18,1	68,0%	914	10,9	72,1%	409	32,3	75,4%	349	34,8	74,0%	172	185,9	87,2%	.	.	.	.	.	.	.	.	.
8 trajectories with quadratic polynomial functional form	-46110,69740	P<.001	344	33,8	77,2%	2195	6,2	81,9%	426	19,8	68,3%	756	10,4	65,5%	211	32,1	62,5%	351	37,0	75,4%	359	34,9	74,0%	161	208,0	87,9%	.	.	.	.	.	.
9 trajectories with quadratic polynomial functional form	-46136,85120	P<.001	139	66,9	64,5%	220	32,1	63,7%	2110	7,0	83,6%	910	11,1	72,0%	471	18,6	69,3%	101	99,1	73,3%	514	19,5	68,8%	166	66,8	73,7%	172	205,6	88,5%	.	.	.
10 trajectories with quadratic polynomial functional form	-46119,63710	P<.001	270	38,3	70,5%	99	25,2	55,5%	2058	5,6	77,6%	481	18,4	68,5%	201	32,6	62,6%	437	28,5	73,9%	356	35,8	74,1%	693	9,9	62,9%	141	129,9	80,7%	67	319,0	82,0%
2 trajectories with cubic polynomial functional form	-47132,85060	0,10	3391	8,9	95,4%	1412	24,3	91,3%	.	.	.	.	.	.	.	.	.	.	.	.	.	.	.	.	.	.	.	.	.	.	.	.
3 trajectories with cubic polynomial functional form	-46622,50960	0,25	2593	9,5	91,6%	1811	9,7	85,5%	399	79,4	88,6%	.	.	.	.	.	.	.	.	.	.	.	.	.	.	.	.	.	.	.	.	.
4 trajectories with cubic polynomial functional form	-46364,46230	P<.001	2661	10,6	92,6%	541	23,1	76,8%	1204	11,8	79,7%	397	86,2	88,8%	.	.	.	.	.	.	.	.	.	.	.	.	.	.	.	.	.	.
5 trajectories with cubic polynomial functional form	-46182,06140	P<.001	1216	11,9	80,8%	2079	5,3	78,9%	795	20,6	80,5%	452	29,8	77,1%	261	132,6	88,4%	.	.	.	.	.	.	.	.	.	.	.	.	.	.	.
6 trajectories with cubic polynomial functional form	-46090,75230	P<.001	707	17,7	77,7%	812	13,0	73,7%	2073	5,4	78,5%	515	30,7	78,5%	529	26,7	77,3%	167	208,5	88,7%	.	.	.	.	.	.	.	.	.	.	.	.
7 trajectories with cubic polynomial functional form	-46025,40100	P<.001	1808	11,3	86,6%	1206	7,1	69,8%	410	26,5	73,7%	508	30,8	78,6%	318	26,5	67,7%	368	37,1	75,3%	185	215,4	89,6%	.	.	.	.	.	.	.	.	.
8 trajectories with cubic polynomial functional form	-45955,75600	P<.001	496	24,1	75,3%	2093	6,5	82,0%	425	18,2	65,6%	393	29,2	73,4%	635	15,8	70,9%	305	49,0	77,3%	313	48,3	77,5%	143	254,5	88,9%	.	.	.	.	.	.
9 trajectories with cubic polynomial functional form	-46025,62460	P<.001	808	13,7	71,1%	317	22,6	65,7%	1501	5,6	67,6%	628	16,4	72,0%	271	22,4	69,5%	350	40,1	76,1%	300	26,5	66,1%	460	29,6	75,6%	168	209,8	88,7%	.	.	.
10 trajectories with cubic polynomial functional form	-45911,83000	P<.001	327	41,4	76,9%	229	32,1	69,0%	1888	6,4	78,0%	771	12,1	69,0%	388	31,6	74,9%	290	32,0	69,3%	270	41,2	71,8%	143	77,9	73,1%	331	47,8	78,3%	166	228,6	89,0%

BIC: Bayesian Information Criterion based on the number of observations (to maximize) / X^2: P value for Chi-square test of nullity of higher degree terms / n: number of patients

© 2022 Charles C et al. *JAMA Network Open.*

eTable 7. Epidemiological Model Outcomes

**Variables**[a]	**P value**[b]
Having children	0.002
Occupational class	0.004
Household income	<.001
Familial history of breast cancer	<.001
Former psychiatric hospitalizations	<.001
Cancer stage	0.040
Body Mass Index	<.001
Smoking status	<.001
Level of physical activity	<.001

[a] Variables included in the model as covariates were: age (< 40; [40-75]; > 75), marital status, having children, level of education, working status, occupational category, household income, personal and familial history of cancer, former psychiatric hospitalizations, tumor stage and subtype, Charlson comorbidity index, menopausal status, Body Mass Index, smoking status, alcohol consumption, level of physical activity.

[b] Wald nullity test

© 2022 Charles C et al. *JAMA Network Open.*

eTable 8. Clinical Model Outcomes

**Variables**[a]	**P value**[b]
Pain	0.05
Fatigue	<.001
HADS-D score (depression)	<.001

[a] Variables included in the model as covariates were: pain, fatigue, insomnia, satisfaction with body image, and level of depression (HADS-D, continuous score) at baseline.
[b] Wald nullity test

© 2022 Charles C et al. *JAMA Network Open.*

eTable 9. Description of Baseline Patients' Characteristics Based on Trajectory Affiliation

Characteristics	Non-cases	Delayed occurrence	Intermediate worsening	Intermediate improving	Remission	Stable depression	All
	**N = 2634 (54.84%)**	**N = 200 (4.16%)**	**N = 1076 (22.40%)**	**N = 480 (9.99%)**	**N = 261 (5.43%)**	**N = 152 (3.16%)**	**N = 4803**
**Demographic factors**							
**Age**							
Mean (SD)	56.4 (11.2)	57.5 (10.5)	56.3 (11.3)	56.2 (11.4)	52.9 (11.1)	56.8 (11.0)	56.2 (11.2)
Range	[22.0-88.0]	[29.0-85.0]	[26.0-86.0]	[30.0-88.0]	[25.0-82.0]	[34.0-84.0]	[22.0-88.0]
**Marital status**							
Partnered	1915 (72.7%)	135 (68.0%)	746 (69.3%)	325 (67.7%)	180 (69.0%)	98 (64.5%)	3400 (70.8%)
Not partnered	614 (23.3%)	52 (26.0%)	274 (25.5%)	127 (26.5%)	73 (28.0%)	41 (27.0%)	1181 (24.6%)
Missing	105 (4.0%)	12 (6.0%)	56 (5.2%)	28 (5.8%)	8 (3.1%)	13 (8.6%)	222 (4.6%)
**Dependent children**							
0	1316 (50.0%)	89 (44.5%)	503 (46.7%)	228 (47.5%)	100 (38.3%)	70 (46.1%)	2306 (48.0%)
1+	974 (37.0%)	80 (40.0%)	386 (35.9%)	180 (37.5%)	132 (50.6%)	51 (33.6%)	1803 (37.5%)
Missing	344 (13.1%)	31 (15.5%)	187 (17.4%)	72 (15.0%)	29 (11.1%)	31 (20.4%)	694 (14.4%)
**Social factors**							
**Level of education**							
Primary school	323 (12.3%)	43 (21.5%)	160 (14.9%)	91 (19.0%)	24 (9.2%)	32 (21.1%)	673 (14.0%)
High school	1156 (43.9%)	100 (50.0%)	507 (47.1%)	237 (49.4%)	125 (47.9%)	74 (48.7%)	2199 (45.8%)
College or higher	1088 (41.3%)	52 (26.0%)	372 (34.6%)	136 (28.3%)	105 (40.2%)	40 (26.3%)	1793 (37.3%)
Missing	67 (2.5%)	5 (2.5%)	37 (3.4%)	16 (3.3%)	7 (2.7%)	6 (3.9%)	138 (2.9%)
**Working status**							
Active	1373 (52.1%)	100 (50.0%)	572 (53.2%)	245 (51%)	161 (61.7%)	76 (50.0%)	2527 (52.6%)
Inactive	1230 (46.7%)	98 (49.0%)	488 (45.4%)	231 (48.1%)	97 (37.2%)	74 (48.7%)	2218 (46.2%)
Missing	31 (1.2%)	2 (1.0%)	16 (1.5%)	4 (0.8%)	3 (1.1%)	2 (1.3%)	58 (1.2%)
**Occupational category**							
Self-employed	177 (6.7%)	15 (7.5%)	83 (7.7%)	37 (7.7%)	18 (6.9%)	12 (7.9%)	342 (7.1%)
Manual workers	165 (6.3%)	30 (15%)	96 (8.9%)	58 (12.1%)	13 (5%)	16 (10.5%)	378 (7.9%)
Technicians	626 (23.8%)	29 (14.5%)	244 (22.7%)	100 (20.8%)	71 (27.2%)	23 (15.1%)	1093 (22.8%)
Clerks	860 (32.6%)	77 (38.5%)	395 (36.7%)	195 (40.6%)	104 (39.8%)	68 (44.7%)	1699 (35.4%)

© 2022 Charles C et al. *JAMA Network Open.*

Manager, intellectual profession	661 (25.1%)	32 (16%)	201 (18.7%)	66 (13.8%)	43 (16.5%)	26 (17.1%)	1029 (21.4%)
Undefined	84 (3.2%)	12 (6%)	25 (2.3%)	10 (2.1%)	5 (1.9%)	2 (1.3%)	138 (2.9%)
Missing	61 (2.3%)	5 (2.5%)	32 (3%)	14 (2.9%)	7 (2.7%)	5 (3.3%)	124 (2.6%)

**Characteristics**	**Non cases**	**Delayed occurrence**	**Intermediate worsening**	**Intermediate improving**	**Remission**	**Stable depression**	**All**
	**N = 2634 (54.84%)**	**N = 200 (4.16%)**	**N = 1076 (22.40%)**	**N = 480 (9.99%)**	**N = 261 (5.43%)**	**N = 152 (3.16%)**	**N = 4803**
**Household income** (euros per month)							
< 1500	271 (10.3%)	35 (17.5%)	158 (14.7%)	88 (18.3%)	31 (11.9%)	37 (24.3%)	620 (12.9%)
[1500-3000]	896 (34.0%)	86 (43.0%)	413 (38.4%)	198 (41.3%)	95 (36.4%)	66 (43.4%)	1754 (36.5%)
> 3000	1221 (46.4%)	56 (28.0%)	397 (36.9%)	150 (31.3%)	107 (41.0%)	36 (23.7%)	1967 (41.0%)
Missing	246 (9.3%)	23 (11.5%)	108 (10.0%)	44 (9.2%)	28 (10.7%)	13 (8.6%)	462 (9.6%)
**Medical history**							
**Personal history of cancer**							
Yes	222 (8.4%)	17 (8.5%)	82 (7.6%)	43 (9.0%)	15 (5.7%)	21 (13.8%)	400 (8.3%)
No	2386 (90.6%)	181 (90.5%)	978 (90.9%)	434 (90.4%)	243 (93.1%)	130 (85.5%)	4352 (90.6%)
Missing	26 (1.0%)	2 (1.0%)	16 (1.5%)	3 (0.6%)	3 (1.1%)	1 (0.7%)	51 (1.1%)
**Familial history of breast cancer (1st/2nd degree)**							
0	1452 (55.1%)	106 (53.0%)	542 (50.4%)	254 (52.9%)	142 (54.4%)	83 (54.6%)	2579 (53.7%)
1+	1136 (43.1%)	92 (46.0%)	514 (47.8%)	203 (42.3%)	116 (44.4%)	62 (40.8%)	2123 (44.2%)
Missing	46 (1.7%)	2 (1.0%)	20 (1.9%)	23 (4.8%)	3 (1.1%)	7 (4.6%)	101 (2.1%)
**Familial history of cancer (1st/2nd)**							
0	1378 (52.3%)	95 (47.5%)	545 (50.7%)	235 (49.0%)	134 (51.3%)	75 (49.3%)	2462 (51.3%)
1+	1194 (45.3%)	102 (51.0%)	505 (46.9%)	227 (47.3%)	125 (47.9%)	71 (46.7%)	2224 (46.3%)
Missing	62 (2.4%)	3 (1.5%)	26 (2.4%)	18 (3.8%)	2 (0.8%)	6 (3.9%)	117 (2.4%)
**Former psychiatric hospitalizations**							
Yes	41 (1.6%)	12 (6.0%)	34 (3.2%)	23 (4.8%)	9 (3.4%)	12 (7.9%)	131 (2.7%)
No	2552 (96.9%)	180 (90%)	1016 (94.4%)	445 (92.7%)	246 (94.3%)	139 (91.4%)	4578 (95.3%)
Missing	41 (1.6%)	8 (4.0%)	26 (2.4%)	12 (2.5%)	6 (2.3%)	1 (0.7%)	94 (2.0%)

© 2022 Charles C et al. *JAMA Network Open.*

Former psychiatric disorders							
Yes	302 (11.5%)	49 (24.5%)	199 (18.5%)	109 (22.7%)	54 (20.7%)	55 (36.2%)	768 (16.0%)
No	2238 (85.0%)	146 (73.0%)	838 (77.9%)	354 (73.8%)	202 (77.4%)	96 (63.2%)	3874 (80.7%)
Missing	94 (3.6%)	5 (2.5%)	39 (3.6%)	17 (3.5%)	5 (1.9%)	1 (0.7%)	161 (3.4%)

Characteristics	Non cases	Delayed occurrence	Intermediate worsening	Intermediate improving	Remission	Stable depression	All
	**N = 2634 (54.84%)**	**N = 200 (4.16%)**	**N = 1076 (22.40%)**	**N = 480 (9.99%)**	**N = 261 (5.43%)**	**N = 152 (3.16%)**	**N = 4803**
**Clinical factors**							
**Tumor stage**							
Stage I	1404 (53.3%)	81 (40.5%)	553 (51.4%)	213 (44.4%)	123 (47.1%)	67 (44.1%)	2441 (50.8%)
Stage II	990 (37.6%)	97 (48.5%)	432 (40.1%)	208 (43.3%)	113 (43.3%)	67 (44.1%)	1907 (39.7%)
Stage III	239 (9.1%)	21 (10.5%)	90 (8.4%)	59 (12.3%)	25 (9.6%)	18 (11.8%)	452 (9.4%)
Missing	1 (0.04%)	1 (0.5%)	1 (0.1%)	.	.	.	3 (0.1%)
**Tumor subtype**							
HR+ HER2+	269 (10.2%)	27 (13.5%)	119 (11.1%)	49 (10.2%)	31 (11.9%)	11 (7.2%)	506 (10.5%)
HR+ HER2-	2005 (76.1%)	158 (79.0%)	821 (76.3%)	357 (74.4%)	201 (77%)	121 (79.6%)	3663 (76.3%)
HR- HER2+	119 (4.5%)	4 (2.0%)	41 (3.8%)	18 (3.8%)	9 (3.4%)	6 (3.9%)	197 (4.1%)
HR- HER2-	220 (8.4%)	11 (5.5%)	86 (8.0%)	54 (11.3%)	20 (7.7%)	14 (9.2%)	505 (8.4%)
Missing	21 (0.8%)	.	9 (0.8%)	2 (0.4%)	.	.	32 (0.7%)
**Charlson comorbidity index**							
0	2007 (76.2%)	138 (69.0%)	806 (74.9%)	323 (67.3%)	191 (73.2%)	102 (67.1%)	3567 (74.3%)
1	236 (9.0%)	24 (12.0%)	97 (9.0%)	63 (13.1%)	24 (9.2%)	14 (9.2%)	458 (9.5%)
2+	218 (8.3%)	22 (11.0%)	96 (8.9%)	51 (10.6%)	28 (10.7%)	19 (12.5%)	434 (9.0%)
Missing	173 (6.6%)	16 (8.0%)	77 (7.2%)	43 (9.0%)	18 (6.9%)	17 (11.2%)	344 (7.2%)
**Menopausal status**							
Premenopausal	945 (35.9%)	61 (30.5%)	399 (37.1%)	181 (37.7%)	123 (47.1%)	46 (30.3%)	1755 (36.5%)
Postmenopausal	1641 (62.3%)	134 (67.0%)	658 (61.2%)	292 (60.8%)	131 (50.2%)	103 (67.8%)	2959 (61.6%)
Missing	48 (1.8%)	5 (2.5%)	19 (1.8%)	7 (1.5%)	7 (2.7%)	3 (2.0%)	89 (1.9%)
**Body Mass Index**							
Underweight <18.5	56 (2.1%)	4 (2.0%)	25 (2.3%)	9 (1.9%)	8 (3.1%)	2 (1.3%)	104 (2.2%)
Normal [18.5–25[	1421 (53.9%)	75 (37.5%)	467 (43.4%)	198 (41.3%)	139 (53.3%)	68 (44.7%)	2368 (49.3%)
Overweight [25-30[	723 (27.4%)	65 (32.5%)	351 (32.6%)	146 (30.4%)	66 (25.3%)	44 (28.9%)	1395 (29%)

© 2022 Charles C et al. *JAMA Network Open.*

Obese ≥30	427 (16.2%)	56 (28.0%)	228 (21.2%)	126 (26.3%)	47 (18.0%)	36 (23.7%)	920 (19.2%)
Missing	7 (0.3%)	.	5 (0.5%)	1 (0.2%)	1 (0.4%)	2 (1.3%)	16 (0.3%)
**Symptoms**							
**Pain**							
< 40	2490 (94.5%)	172 (86.0%)	956 (88.8%)	372 (77.5%)	211 (80.8%)	93 (61.2%)	4294 (89.4%)
≥ 40	134 (5.1%)	28 (14.0%)	118 (11%)	106 (22.1%)	48 (18.4%)	59 (38.8%)	493 (10.3%)
Missing	10 (0.4%)	.	2 (0.2%)	2 (0.4%)	2 (0.8%)	.	16 (0.3%)

Characteristics	Non cases	Delayed occurrence	Intermediate worsening	Intermediate improving	Remission	Stable depression	All
	**N = 2634 (54.84%)**	**N = 200 (4.16%)**	**N = 1076 (22.40%)**	**N = 480 (9.99%)**	**N = 261 (5.43%)**	**N = 152 (3.16%)**	**N = 4803**
**Fatigue**							
< 40	2317 (88.0%)	131 (65.5%)	769 (71.5%)	237 (49.4%)	150 (57.5%)	45 (29.6%)	3649 (76.0%)
≥ 40	311 (11.8%)	69 (34.5%)	305 (28.3%)	242 (50.4%)	109 (41.8%)	107 (70.4%)	1143 (23.8%)
Missing	6 (0.2%)	.	2 (0.2%)	1 (0.2%)	2 (0.8%)	.	11 (0.2%)
**Insomnia**							
Yes	1841 (69.9%)	163 (81.5%)	829 (77.0%)	418 (87.1%)	228 (87.4%)	130 (85.5%)	3609 (75.1%)
No	768 (29.2%)	37 (18.5%)	236 (21.9%)	57 (11.9%)	30 (11.5%)	21 (13.8%)	1149 (23.9%)
Missing	25 (0.9%)	.	11 (1.0%)	5 (1.0%)	3 (1.1%)	1 (0.7%)	45 (0.9%)
**Anxiety (HADS-A)**							
Mean (SD)	7.6 (3.7)	9.2 (3.9)	9.3 (3.7)	12.5 (3.5)	13.2 (3.8)	13.6 (4.0)	9.0 (4.2)
Range	[0.0-20.0]	[1.0-19.0]	[0.0-20.0]	[2.0-21.0]	[0.0-21.0]	[1.0-20.0]	[0.0-21.0]
[0-7]	1363 (51.7%)	76 (38.0%)	361 (33.6%)	39 (8.1%)	18 (6.9%)	11 (7.2%)	1868 (38.9%)
[8-10]	722 (27.4%)	57 (28.5%)	327 (30.4%)	100 (20.8%)	47 (18.0%)	26 (17.1%)	1279 (26.6%)
≥ 11	545 (20.7%)	66 (33.0%)	388 (36.1%)	341 (71.0%)	196 (75.1%)	115 (75.7%)	1651 (34.4%)
Missing	4 (0.2%)	1 (0.5%)	.	.	.	.	5 (0.1%)
**Depression (HADS-D)**							
Mean (SD)	2.2 (2.0)	4.3 (2.2)	4.1 (2.2)	9.2 (2.3)	10.2 (2.2)	12.5 (2.9)	4.2 (3.7)
Range	[0.0-8.0]	[0.0-9.0]	[0.0-10.0]	[3.0-17.0]	[7.0-19.0]	[6.0-21.0]	[0.0-21.0]
[0-4]	2253 (85.5%)	100 (50.0%)	592 (55.0%)	4 (0.8%)	.	.	2949 (61.4%)
[5-7]	358 (13.6%)	88 (44.0%)	439 (40.8%)	100 (20.8%)	8 (3.1%)	4 (2.6%)	997 (20.8%)
[8-10]	23 (0.9%)	12 (6.0%)	45 (4.2%)	248 (51.7%)	161 (61.7%)	34 (22.4%)	523 (10.9%)
≥ 11	.	.	.	128 (26.7%)	92 (35.2%)	114 (75.0%)	334 (7.0%)
Missing	4 (0.2%)	1 (0.5%)	.	.	.	.	5 (0.1%)

© 2022 Charles C et al. *JAMA Network Open.*

**Depression (BDI-SF)**							
Mean	1.9 (2.2)	4.8 (3.5)	3.8 (3.1)	8.1 (5.0)	6.8 (4.0)	13.0 (6.5)	3.7 (4.1)
Range	[0.0-15.0]	[0.0-18.0]	[0.0-25.0]	[0.0-38.0]	[0.0-20.0]	[0.0-36.0]	[0.0-38.0]
[0-3]	2137 (82%)	84 (42.0%)	583 (54.7%)	74 (15.6%)	50 (19.4%)	3 (2.0%)	2931 (61.7%)
[4-7]	399 (15.3%)	75 (37.5%)	354 (33.2%)	188 (39.6%)	118 (45.7%)	31 (21.1%)	1165 (24.5%)
[8-15]	70 (2.7%)	40 (20.0%)	125 (11.7%)	174 (36.6%)	81 (31.4%)	72 (49.0%)	562 (11.8%)
≥ 16	0 (0.0%)	1 (0.5%)	3 (0.3%)	39 (8.2%)	9 (3.5%)	41 (27.9%)	93 (2.0%)
Missing	28 (1.2%)	.	11 (1.0%)	5 (1.0%)	3 (1.1%)	5 (3.3%)	52 (1.1%)

Characteristics	Non cases	Delayed occurrence	Intermediate worsening	Intermediate improving	Remission	Stable depression	All
	**N = 2634 (54.84%)**	**N = 200 (4.16%)**	**N = 1076 (22.40%)**	**N = 480 (9.99%)**	**N = 261 (5.43%)**	**N = 152 (3.16%)**	**N = 4803**
**Lifestyle factors**							
**Alcohol consumption**							
Daily	341 (12.9%)	23 (11.5%)	154 (14.3%)	72 (15.0%)	30 (11.5%)	26 (17.1%)	646 (13.4%)
Less than daily	2222 (84.4%)	170 (85.0%)	890 (82.7%)	394 (82.1%)	226 (86.6%)	119 (78.3%)	4021 (83.7%)
Missing	71 (2.7%)	7 (3.5%)	32 (3.0%)	14 (2.9%)	5 (1.9%)	7 (4.6%)	136 (2.8%)
**Smoking status**							
Non smoker	1690 (64.2%)	121 (60.5%)	635 (59.0%)	280 (58.3%)	148 (56.7%)	70 (46.1%)	2944 (61.3%)
Former smoker	573 (21.8%)	40 (20.0%)	217 (20.2%)	99 (20.6%)	66 (25.3%)	36 (23.7%)	1031 (21.5%)
Smoker	325 (12.3%)	37 (18.5%)	206 (19.1%)	97 (20.2%)	44 (16.9%)	44 (28.9%)	753 (15.7%)
Missing	46 (1.7%)	2 (1.0%)	18 (1.7%)	4 (0.8%)	3 (1.1%)	2 (1.3%)	75 (1.6%)
**Physical activity (MET-mn per week)**							
< 600	997 (37.9%)	92 (46%)	437 (40.6%)	226 (47.1%)	123 (47.1%)	91 (59.9%)	1966 (40.9%)
≥ 600	1620 (61.5%)	104 (52%)	627 (58.3%)	243 (50.6%)	134 (51.3%)	58 (38.2%)	2786 (58%)
Missing	17 (0.6%)	4 (2.0%)	12 (1.1%)	11 (2.3%)	4 (1.5%)	3 (2.0%)	51 (1.1%)
**Body image (BRBI)**							
Mean (SD)	93.4 (13.5)	85.8 (21.6)	87.6 (19.2)	76.8 (26.8)	78.5 (24.1)	63.2 (31.1)	88.4 (19.7)
< 100	770 (29.2%)	90 (45.0%)	467 (43.4%)	300 (62.5%)	157 (60.2%)	112 (73.7%)	1896 (39.5%)
= 100	1792 (68.0%)	104 (52.0%)	571 (53.1%)	166 (34.6%)	91 (34.9%)	34 (22.4%)	2758 (57.4%)
Missing	72 (2.7%)	6 (3.0%)	38 (3.5%)	14 (2.9%)	13 (5.0%)	6 (3.9%)	149 (3.1%)

© 2022 Charles C et al. *JAMA Network Open.*

SD, Standard Deviation; HR, Hormone Receptor; HER2, Human Epidermal Growth Factor Receptor 2; HADS, Hospital Anxiety and Depression Scale; BDI-SF, Beck Depression Scale Short-Form; MET-mn, Metabolic-equivalent of task-minute; BRBI, Breast-related body image, 4-item subscale of EORTC QLQ-C3.

© 2022 Charles C et al. *JAMA Network Open.*
